# Diagnostic and prognostic value of a disintegrin and metalloproteinase-17 in patients with gliomas

**DOI:** 10.3892/ol.2014.2582

**Published:** 2014-10-01

**Authors:** BIN WU, LONGGUI SHA, YONG WANG, WEI XU, YANG YU, FANG FENG, CAIXING SUN, LIANG XIA

**Affiliations:** 1 Department of Neurosurgery, Zhejiang Cancer Hospital, Hangzhou, Zhejiang 310022, P.R. China; 2 Department of Neurosurgery, Shanghai Pudong Hospital, Fudan University Pudong Medical Center, Pudong, Shanghai 200120, P.R. China; 3 Department of Neurosurgery, The Second Affiliated Hospital of Nantong University, Nantong, Jiangsu 226001, P.R. China

**Keywords:** ADAM17, expression, prognostic value, glioma

## Abstract

A disintegrin and metalloproteinase-17 (ADAM17) has been shown to regulate numerous proteins involved in the cell cycle, as well as tumor oncogenes. The expression pattern of ADAM17 in glioma patients, however, is unclear. In the present study, the expression pattern and prognostic significance of ADAM17 was investigated in patients with glioma. A total of 60 glioma specimens and eight normal control samples were obtained. Immunohistochemical and western blot analyses were used to examine the expression of ADAM17. In addition, the association of ADAM17 expression with the clinicopathological parameters and the survival rates of the glioma patients was analyzed. The results showed that ADAM17 was upregulated in the high-grade glioma tissues compared with that in the low-grade and normal brain tissues of the glioma patients, and that the level increased with ascending World Health Organization tumor grade (P<0.05). Furthermore, the survival rate of the patients with ADAM17-positive tumors was lower compared with the patients with ADAM17-negative tumors. These results indicated that the overexpression of ADAM17 was correlated with a high tumor grade and a poor prognosis in patients with glioma. ADAM17 may have an important oncogenic function in glioma progression, and is a potential diagnostic and therapeutic target.

## Introduction

Glioma is one of the most common nervous system malignancies. According to the Central Brain Tumor Registry of the United States, 70% of primary malignant brain tumors are gliomas, for which the annual incidence rate is ~5/100,000, with >14,000 new cases each year (1). Although the diagnosis and treatment of glioma has progressed, the overall prognosis of glioma patients remains poor, and the five-year survival rate is ~25% (2). The difficulties in treating malignant glioma is partly due to its malignant biological properties, such as its over-proliferation and high invasiveness (3). The occurrence and development of tumors is the integral result of multiple genes, factors, steps and evolutionary stages. The activation of oncogenes and inactivation or mutation of tumor suppressor genes is associated with tumorigenesis (1). As the underlying mechanism of glioma remains unclear, it is important to investigate the development and invasive behavior of glioma on the genetic level. An increasing number of studies are now aiming to identify the key gene targets of glioma, and to find a mechanism by which to reverse their malignant behavior (4).

The a disintegrin and metalloproteinase-17 (ADAM) superfamily consists of a group of transmembrane multi-domain secretary proteins, which release important ligands, such as tumor necrosis factor α (TNF-α) and epidermal growth factor (EGF), thereby promoting the formation and progression of tumors (5,6). ADAM17, a member of the ADAM family, is also known as tumor necrosis factor-α converting enzyme (TACE) (7). The primary function of ADAM17 is to hydrolyze and release protein precursor molecules on the cell surface, resulting in lateral activation of cell surface molecules in cell signaling pathways, thereby altering signal transduction (8). Recent studies have shown that ADAM17 is highly expressed in non-small cell lung cancer, breast cancer and other malignant tumors, and is associated with the degree of malignancy (9–11). Additionally, ADAM17 has been shown to function as an oncogene, promoting U87 glioblastoma stem cell migration and invasion (12). However, whether ADAM17 is highly expressed in glioma is not known. The present study analyzed ADAM17 expression in normal and glioma brain tissue, and investigated the association between the ADAM17 expression level and the malignancy and prognosis observed in glioma patients.

In order to study the role of ADAM17 expression in glioma, the expression of the protein was analyzed in 60 patients with glioma and in eight control cases (patients with traumatic brain injury) by western blotting and immunohistochemistry (IHC). The association between ADAM17, the malignancy of the glioma and the clinicopathological factors were determined. The association between ADAM17 and prognosis was also analyzed using the Kaplan-Meier method.

## Materials and methods

### Tumor specimens

A total of 60 glioma patients who were treated in the Second Affiliated Hospital of Nantong University (Nantong, China) between 2006 and 2013 were included in the present study. The cohort consisted of 36 males and 24 females, with a mean age of 45 years (5–85 years). In 18 cases, the tumor was located in the frontal lobe, in 23 cases it was located in the temporal lobe and in 19 cases it was located in other regions. The tumor diameter was measured during surgery. All patients underwent a subtotal tumor removal without pre-operative radiotherapy, and then underwent post-operative radiotherapy (60 Gy for one month with single doses of 1.8–2.0 Gy) and chemotherapy with Semustine (200–225 mg/m^2^ every six weeks for six months). Patients that succumbed to other diseases or accidents were excluded from the study. All patients underwent a pre-operative functional status assessment according to the Karnofsky score (KPS) for accurate scoring. A total of 33 cases presented with a KPS score of <80, while 27 cases presented with a score of >80, indicating their inability to perform normal activities. According to the 2007 World Health Organization (WHO) classification of tumors of the central nervous system (13), all gliomas were subjected to histological grading as follows: 25 cases formed the low-grade malignant carcinoma group (grades I and II) and 35 cases formed the high-grade malignant carcinoma group (grades III and IV). All nine grade I tumors were pilocytic astrocytoma. The 16 grade II cases were divided into 12 cases of diffuse astrocytoma, three cases of oligodendrocyte cell tumors and another one case of ependymoma. A total of 19 grade III tumors were anaplastic astrocytomas, and the 16 grade IV tumors were glioblastoma multiforme tumors. The surgically resected brain tissue of eight trauma patients admitted to the hospital within the same period was used as a control group; all cases were pathologically confirmed to be without gliosis.

All specimens were divided into two parts; one part was fixed with 10% neutral formalin and embedded, from which paraffin sections were cut to a thickness of 5 μm, while the other part was immediately frozen at −80°C. Informed consent for use of the specimens was provided by the family members of the patients or the patients themselves. All experimental procedures were approved by the Hospital Ethics Committee of the Second Affiliated Hospital of Nantong University (Nantong, China).

### Reagents

The ADAM17 monoclonal mouse anti-human antibody was provided by Abcam (Cambridge, UK), and the horseradish peroxidase-conjugated donkey anti-mouse immunoglobulin G (IgG, H+L) and Polymerization Peroxidase-labeled Goat anti-Mouse IgG Western Blot kit were purchased from the Beyotime Institute of Biotechnology (Jiangsu, China). The anti-β-actin monoclonal antibody and Biotinylated Rat Anti-mouse IgG IHC kit was obtained from Boster Biological Technology, Ltd. (Wuhan, China), and TRIzol was obtained from Invitrogen Life Technologies (Carlsbad, CA, USA). The reverse transcription and quantitative polymerase chain reaction (PCR) kits were obtained from Fermentas (Waltham, MA, USA).

### Methods

#### Western blotting

Tissue lysates were homogenized and centrifuged at 13,000 × g for 15 min at 4°C. The supernatants were collected, and the protein concentrations were determined using a Bicinchoninic Acid Protein Assay kit (Pierce Biotechnology, Inc., Rockford, IL, USA). An equal amount (10 μg) of each protein sample was loaded onto 10% SDS-PAGE gels, transferred to pure nitrocellulose membranes (PerkinElmer Life Sciences, Boston, MA, USA), and blocked with 5% skimmed milk in Tris-buffered saline Tween-20 solution. The membranes were split and incubated with rabbit polyclonal anti-human ADAM17 primary antibody (1:1,000) at 4°C overnight. The membranes were subsequently incubated with anti-rabbit secondary antibodies (1:4,000) at room temperature for 1 h. Enhanced chemiluminescence (ECL) was performed using an ECL Western Blotting Detection kit (Pierce Biotechnology, Inc.). The western blotting band density was analyzed using Quantity One software (Bio-Rad, Hercules, CA, USA), and then normalized to the values obtained for β-actin.

#### IHC

Paraffin sections were dipped twice into xylene for 10 min to remove the paraffin. The xylene was then removed with a graded alcohol series (100, 95 and 70%). The sections was rinsed with deionized water for 5 min followed by treatment with 3% H_2_O_2_ for inactivation of endogenous peroxidases. Next, the sections were subjected to heat-induced antigen retrieval with 0.01 mm/l phosphate-buffered saline (PBS), and stained using the avidin-biotin-peroxidase complex method with diaminobenzidine as the substrate. The sections were then subjected to hematoxylin and eosin counter staining. Microscopic observations were performed at ×400 magnification in five fields, from which a total of 500 tumor cells were counted. PBS was used, instead of primary antibody, as a negative control. Positive expression was observed as cytoplasmic yellow-, brown- or tan-colored staining. A semi-quantitative analysis was conducted for the staining intensity: No positive cells or <15% positive cells was defined as negative (−); 15 to 25% positive cells, where a pale yellow color was observed, were defined as weakly positive (+); 25 to 50% positive cells, where a brown-yellow color was observed, were defined as moderately positive (++); and >50% positive cells, with a dark brown, yellow or brown color, were defined as strongly positive (+++).

### Statistical analysis

All data are expressed as the mean ± standard error of the mean. Differences in the IHC results were assessed using a χ^2^ test and a rank correlation analysis. Western blotting results were assessed using a one-way analysis of variance, a post-hoc analysis and a rank correlation analysis. P<0.05 was considered to indicate a statistically significant difference.

## Results

### Western blot analysis of ADAM17 protein expression in glioma tissues of different grades

**Figure 1:**
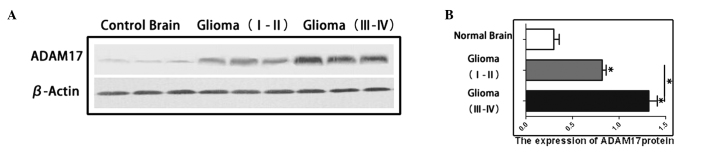
ADAM17 protein expression significantly increases with ascending glioma grade. (A) Expression of ADAM17 protein level in different grades of glioma. (B) Graphical representation of the ADAM17 protein level expression profiles. Data are presented as the mean ± standard error, ^*^P<0.05, III–IV vs. I–II, III–IV vs. normal, and I–II vs. normal. ADAM17, a disintegrin and metalloproteinase-17.

Western blotting showed that the expression of ADAM17 in the high-grade (WHO III–IV; 1.292±0.140) and low-grade (WHO I–II; 0.823±0.101) glioma groups were significantly higher compared with the control brain tissue (0.325±0.068). The expression level of ADAM17 in the high- and low-grade gliomas was significantly higher compared with that of the control brain tissue (P<0.05) (Fig. 1).

### IHC staining of ADAM17 in glioma tissues of different grades

**Figure 2:**
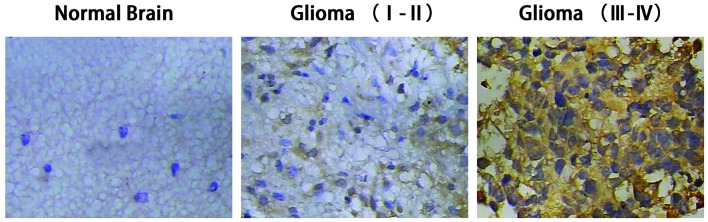
Immunohistochemical analysis of ADAM17 immunoreactivity in normal brain and glioma tissues (magnification, ×400). Gliomas with high grades exhibited stronger ADAM17 protein expression compared with gliomas with lower grades and the normal brain tissues (P<0.05). ADAM17, a disintegrin and metalloproteinase-17.

The ADAM17 protein expression in the eight control and 60 glioma cases was detected by IHC staining. The results showed that ADAM17 was predominantly located in the cytoplasm and was rarely expressed in the nucleus (Fig. 2). The positive rate of ADAM17 expression in the glioma patients of all grades was 88.33%, whereas it was only 37.5% in the control group. The strong positive expression rate of ADAM17 in the control, low-grade glioma and high-grade glioma groups was 0, 0.4 and 51.43%, respectively. The negative expression rate of ADAM17 in the control group was 62.5%, whereas it was only 0.29% in the high-grade glioma group (Table I). These results indicated that ADAM17 expression was significantly higher in the high grade glioma group (WHO III–IV) compared with the low-grade glioma (WHO I–II) and control groups.

### Correlation analysis between ADAM17 expression and glioma clinicopathological factors and prognosis

**Figure 3:**
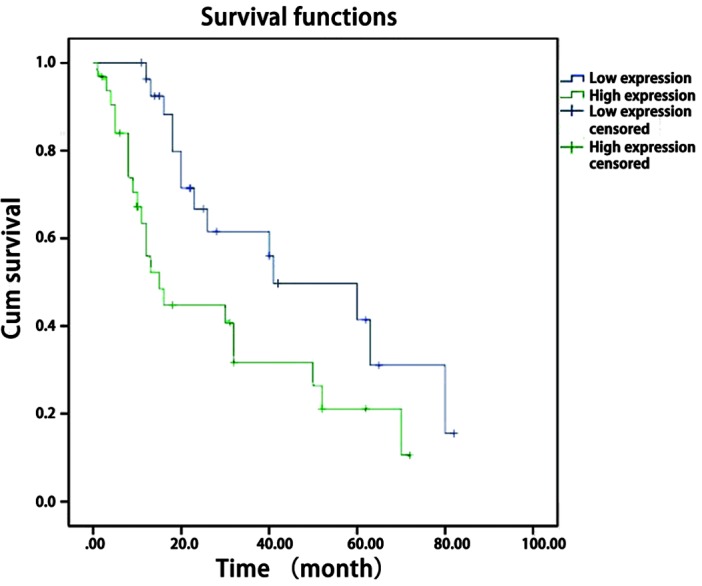
Kaplan-Meier survival curves for ADAM17 expression. The patients with high ADAM17 expression had a significantly worse outcome compared with the patients with low ADAM17 expression (P<0.05). ADAM17, a disintegrin and metalloproteinase-17.

To further determine the association between ADAM17 expression and the clinical prognosis of patients with glioma, the clinical data of 60 glioma patients were analyzed (Table II). The glioma patients were divided into two subgroups, a high expression group (>45% positive cells) and a low expression group (<45% positive cells), according to the IHC ADAM17 expression data. The results showed that ADAM17 expression was not correlated with gender, age, tumor size, location or necrosis. However, ADAM17 expression was significantly associated with the glioma WHO histological grade (P<0.05) (Table II). The prognosis was significantly different in the patients with high ADAM17 expression compared with the patients with low ADAM17 expression. Survival analysis showed that the patients with low ADAM17 expression longer survival times compared with the patients with high ADAM17 expression (P<0.005) (Fig. 3).

## Discussion

Glioma is the most common intracranial malignancy (14). Despite the rapid development of treatment options for glioma in recent years, including minimally invasive neurosurgical techniques, precise positioning radiotherapy technology and chemotherapy drugs that target all aspects of the tumor growth cycle, the high recurrence and low curative rates caused by the invasion of glioma remain problematic (2). The development of molecular biology techniques and an improved understanding of tumor pathogenesis has allowed the use of targeted therapy in the comprehensive treatment of glioma. Molecular targets have included receptors, key genes and regulatory molecules (1), and molecular-targeted drugs have become novel drug treatments for malignant glioma.

ADAMs are a class of membrane-anchored cell surface proteins, which function in proteolysis, the release of bioactive cytokines, cell adhesion, integration, migration and signal transduction (15). ADAM17, a member of the ADAM gene family, has been shown to be highly expressed in various human tumors, reflecting the degree of malignancy, since ADAM17 promotes tumor invasion and metastasis (16). In a study of ADAM17 expression in breast cancer, McGowan *et al* (17) found that the mRNA and protein levels of ADAM17 in breast cancer tissue were positively correlated with the number of lymph node metastases, suggesting that ADAM17 is closely associated with the progression of breast cancer. ADAM17 has been shown to shed ligands of EGFR, such as amphiregulin and TNFα, and subsequently activate EGFR, thereby improving the proliferation and migration of lung cancer cells (8). Kornfeld *et al* (18) reported that ADAM17 could activate EGFR by releasing amphiregulin, thereby enhancing the proliferation and invasion of head and neck squamous carcinoma cells. EGFR activation is a key step in the tumor growth of a variety of carcinomas, and is associated with the malignancy of astrocytoma (19). EGFR has functions in the proliferation, migration, invasion and DNA damage repair processes of glioma cells, and aberrant signal transduction pathways are able to promote the growth, migration, angiogenesis and apoptosis of the tumor cells (20). Additionally, ADAM17 has been shown to function as an oncogene, promoting U87 glioblastoma stem cell migration and invasion (12).

Overall, the present study hypothesized that ADAM17 is highly expressed in glioma, and that its expression is correlated with malignant glioma. By using western blotting and IHC, 60 cases of glioma specimens were compared with 10 cases of control brain tissues to identify the following: i) In the control group, the expression of ADAM17 protein expression was non-detectable or low, whereas it was highly expressed in the glioma groups; the protein expression of ADAM17 increased with an increase in glioma malignancy. ii) ADAM17 expression was significantly associated with malignant gliomas. iii) Survival analysis confirmed that ADAM17 expression was correlated with patient survival time, such that patients with a high expression of ADAM17 had a worse prognosis. ADAM17 may be used as an indicator of glioma prognosis. Given that ADAM17 enhances cell invasion and the degree of malignancy by activating EGFR in a variety of tumor cells, we propose that ADAM17 may have the same role as an oncogene in glioma, however, the underlying mechanism remains to be investigated.

## Figures and Tables

**Table I:** Expression of ADAM17 in glioma tissue and control brain tissue cases.

Group	Negative	Weakly-positive	Moderately-positive	Strongly-positive	Total
Control, n	5	2	1	0	8
Glioma (I–II), n	2	9	13	1	25
Glioma (III–IV), n	1	3	13	18	35

The overall level of ADAM17 expression was significantly higher in the WHO grade III–IV glioma tissues compared with the WHO I–II glioma and normal brain tissues, as analyzed by immunohistochemistry (P<0.05). ADAM17, a disintegrin and metalloproteinase-17; WHO, World Health Organization.

**Table II:** Association between ADAM17 expression and clinicopathological characteristics of gliomas.

		ADAM17 expression		
			
Clinicopathological features	Total, n	Low expression, n	High expression, n	P-value
Gender				
Male	36	17	19	0.653
Female	24	11	13	
Age				
<45	28	13	15	0.589
>45	32	15	17	
Tumor size, cm				
<4	24	9	15	0.185
>4	36	19	17	
Tumor location				0.789
Frontal	18	8	10	
Temporal	23	12	11	
Other	19	8	11	
Necrosis				
Absence	31	12	19	0.154
Presence	29	16	13	
KPS				
<80	33	14	19	0.320
>80	27	14	13	
WHO grade				
I–II	25	18	7	<0.005
III–IV	35	10	25	

ADAM17-positive expression rate was significantly correlated with the WHO classification of glioma grade. KPS, Karnofsky score; WHO, World Health Organization; ADAM17, a disintegrin and metalloproteinase-17; NS, not significant.
